# The programming effects of nutrition‐induced catch‐up growth on gut microbiota and metabolic diseases in adult mice

**DOI:** 10.1002/mbo3.328

**Published:** 2016-01-08

**Authors:** Jia Zheng, Xinhua Xiao, Qian Zhang, Miao Yu, Jianping Xu, Cuijuan Qi, Tong Wang

**Affiliations:** ^1^ Department of EndocrinologyKey Laboratory of EndocrinologyMinistry of HealthPeking Union Medical College HospitalDiabetes Research Center of Chinese Academy of Medical Sciences and Peking Union Medical CollegeBeijing100730China

**Keywords:** Gut microbiota, maternal imbalanced nutrition, metabolism, offspring

## Abstract

Substantial evidence indicated that catch‐up growth could increase the susceptibility to obesity, insulin resistance, and type 2 diabetes mellitus in adulthood. However, investigations into the “programming” effects of catch‐up growth on gut microbiota in the offspring are limited. C57/BL6 mice were fed on either low protein (LP) or normal chow (NC) diet throughout gestation and lactation. Then, the offspring were randomly weaned to either NC or high fat (HF) diet until 32 weeks of age, generating four experimental groups: NC‐NC, NC‐HF, LP‐NC, and LP‐HF. Metabolic parameters and gut microbiota were examined in the offspring. It showed that the NC‐HF and LP‐HF offspring displayed higher body weight (*P *< 0.05), impaired glucose tolerance (*P *< 0.001), and elevated serum lipids (*P *< 0.05) at 32 weeks of age. Both the operational taxonomic units (OTUs) and the Shannon indexes (*P *< 0.05) showed significantly lower microbial diversity in NC‐HF and LP‐HF offspring. There were significant variations in the compositions of gut microbiota in the NC‐HF and LP‐HF offspring, compared with NC‐NC offspring (*P *< 0.05). Furthermore, it indicated *Lactobacillus* percentage was negatively associated with blood glucose concentrations of intraperitoneal glucose tolerance test (*r* = −0.886, *P* = 0.019). In conclusion, catch‐up growth predisposes the offspring to gut microbiota perturbation, obesity, impaired glucose tolerance, insulin resistance, and dyslipidemia. Our study is novel in showing the “programming” effects of nutrition‐induced catch‐up growth on gut microbiota and metabolic diseases in later life.

## Introduction

It is generally believed that catch‐up growth is a physiological adaptation that allows humans and other higher animals to return to their genetically programmed growth trajectory after a period of growth retardation (Crescenzo et al. 2003). However, substantial epidemiological evidence and experimental animal models demonstrate that maternal imbalanced nutrition also has long‐term pathophysiological consequences. More specifically, it can significantly increase the susceptibility to chronic metabolic diseases such as obesity, insulin resistance, and type 2 diabetes mellitus in later life (Cianfarani et al. 1999; Ong et al. 2000; Roseboom et al. 2001; Xiao et al. 2010; Claycombe et al. 2013; Sellayah et al. 2014). These led to the concept of developmental origin of health and diseases (DOHaD), also known as “fetal programming” (Barker 2004). Because of these findings, the link between maternal imbalanced nutrition and later metabolic disorders has become a subject of active research. However, the mechanisms underlying maternal imbalanced nutrition and such diseases remain largely unclear.

Currently, it is widely accepted that gut microbiota can affect many biological functions throughout the body. It has been estimated that the microbes in our bodies collectively amount to 100 trillion bacteria. The microbes can generate a biomass of about 1–2 kg with 2000 distinct species. Furthermore, the total genome of gut microbiota can contain approximately 100‐fold more unique genes than the human genome (Qin et al. 2010). The majority of the microbes residing in the gut are now regarded as essential to many aspects of human physiology. Numerous associations have now been identified that link the metabolic capacity of the microbiota with human nutrition and metabolism (Flint et al. 2012). Increasingly, studies on gut microbiota have demonstrated that a balance in gut microbial communities is critical for maintaining the health of the host (Gu et al. 2013). However, perturbation of these microbial compositions has been suggested to be involved in the pathogenesis of metabolic diseases outside the gut, such as obesity (Ley et al. 2006), diabetes mellitus (Cani et al. 2008), and even nonalcoholic fatty liver disease (Mouzaki et al. 2013).

Postnatal catch‐up growth can occur when offspring that have experienced protein undernutrition are exposed to an energy‐rich diet (Claycombe et al. 2013). Previous studies have indicated that a maternal low‐protein diet together with a postweaning diet high in fat can significantly increase susceptibility to obesity, glucose intolerance, and insulin resistance in the offspring (Claycombe et al. 2013; Sellayah et al. 2014). Most organs including liver, pancreas, skeletal muscle, and adipose tissue appeared to be imprinted by the effects of catch‐up growth (Warner and Ozanne 2010; Berends et al. 2013). There is, however, little information about the influences on gut microbiota, even though it is known that it plays a central role in nutrient metabolism and the maintenance of the health of the host (Lalles 2012). Moreover, investigations into the “programming” effects of maternal imbalanced nutrition on gut microbiota and metabolic diseases in offspring in adulthood are limited. Given the interrelation and interaction among gut microbiota, nutrition, and metabolism, it can be postulated that catch‐up growth‐induced adaptations of the gut microbiota can be associated with an increased risk of developing obesity and type 2 diabetes mellitus in adulthood. In our present work, we aimed at defining the potential effects of maternal imbalanced nutrition on gut microbial communities and metabolism disturbances in the offspring.

## Materials and Methods

### Ethics statement

The protocol was approved by the animal care and use committee of the Peking Union Medical College Hospital (Beijing, China, MC‐08‐6004) and was conducted in compliance with the Guide for the Care and Use of Laboratory Animals, 8th ed, 2011. All surgery was performed under sodium pentobarbital anesthesia, and all efforts were made to minimize suffering.

### Animals and study design

Seven‐week‐old C57BL/6J mice were obtained from the Institute of Laboratory Animal Science, Chinese Academy of Medical Sciences and Peking Union Medical College (Beijing, China, SCXK‐2014‐0107). The animals were raised and kept under SPF conditions (room temperature at 22 ± 2°C; 12 h light/dark cycle). Mice were given ad libitum access to food and sterile water throughout the study period. Before mating, all the mice were randomly fed with NC diet for 1 week for adaptation. Females were housed in separate cages and were cohabitated with a male for mating upon entering proestrus. Females were checked daily for postcopulatory plugs, and the presence of a plug in the morning after mating was taken as day 0.5 day of pregnancy. Then, the pregnant mice were randomly assigned to two groups and were fed on either NC diet or LP diet. More precisely, the NC diet contains 20% protein, whereas the LP diet contains an isoenergetic 8% protein throughout gestation and lactation, as previous literature described (Snoeck et al. 1990). At day 1 after birth, the litter size of each dam was standardized to six pups, to ensure no litter was nutritionally biased. All the offspring were weaned at 3 weeks of age. At weaning, the male offspring of dams fed on either NC diet or LP diet and then weaned to either NC or HF diet until 32 weeks of age. The purified HF diet was composed of 58% of energy as fat, 25.6% carbohydrates, and 16.4% protein, as previously described (Winzell and Ahren 2004). All the diets were produced and purchased by Research Diets (New Brunswick, NJ). Therefore, it generated four experimental groups in the offspring: NC‐NC (*n* = 8), NC‐HF (*n* = 8), LP‐NC (*n* = 8), and LP‐HF (*n* = 8). Male offspring were maintained until the end of the experimental period. The female offspring were not examined in our present study in order to prevent confounding factors related to their hormone profile and estrus cycle. More importantly, the maternal low‐protein diet animal model has been indicated to exhibit the programming effects in a sexually dimorphic manner, which was not the focus of our investigation (Chamson‐Reig et al. 2009; Sohi et al. 2011).

### Serum and sample collections

To obviate any litter effects, the male offspring (*n* = 8 per group) from four different litters (*n* = 2 per litter) were sacrificed at the end of the experimental period. Blood samples (about 3–4 mL) were collected from the intraorbital retrobulbar plexus in 10‐h fasted anesthetized mice. Then, the blood samples were centrifuged at 4000 ***g*** for 10 min at room temperature and serum was stored in aliquots at −80°C. Then ileocecal region contents were quickly removed, snap frozen in liquid nitrogen, and stored at −80°C for further analysis, as previously described (Karlsson et al. 2011).

### Intraperitoneal glucose tolerance test

All the mice were fasted overnight (12 h), weighed and injected intraperitoneally with a bolus of glucose (2 g/kg of body weight). BG (Blood Glucose) levels were measured at 0, 30, 60, and 120 min after glucose challenge from whole tail vein blood using a Contour TS glucometer (Bayer, Japan). The area under the curve (AUC) of intraperitoneal glucose tolerance test (IPGTT) was calculated.

### Serum biochemical parameters analysis

Serum insulin concentrations were measured using an ELISA kit from ALPCO Diagnostics (80‐INSMSU‐E01, Salem, NH) according to the manufacturer's instructions. Insulin resistance was assessed using the homeostasis model assessment of insulin resistance (HOMA‐IR). The HOMA‐IR was calculated as fasting insulin concentration (*μ*U/mL) × fasting glucose concentration (mmol/L)/22.5. The serum cholesterol and triglyceride levels were measured using colorimetric quantitation kits (K603‐100 and K622‐100, Biovision Inc., Mountain View, CA), respectively. Each sample was measured in duplicate.

### Gut microbiota analysis

Microbial diversity was analyzed according to our recent publication (Zheng et al. 2015). More specifically, microbial DNA was extracted using the E.Z.N.A.^®^ DNA Kit (Omega Bio‐tek, Norcross, GA) according to the manufacturer's protocol. Then, the V3–V4 region of the bacterial 16S rRNA gene sequences were amplified by PCR. The primers are as following: 338F 5′‐ACTCCTACGGGAGGCAGCA‐3′ and 806R 5′‐GGACTACHVGGGTWTCTAAT‐3′ where barcode is an N‐base sequence unique to each sample. PCR reactions were performed and the cycle conditions were 95°C for 2 min, followed by 27 cycles at 95°C for 30 sec, 55°C for 30 sec, and 72°C for 45 sec and a final extension at 72°C for 10 min. Amplicons were extracted from 2% agarose gels and purified using the AxyPrep DNA Gel Extraction Kit (Axygen Biosciences, Union City, CA) and then quantified using QuantiFluor^™^‐ST (Promega, San Luis Obispo, CA USA). Raw fastq files were demultiplexed and quality‐filtered using Quantitative Insights Into Microbial Ecology (version 1.17) with the following three criteria: (1) Reads containing ambiguous characters were removed. (2) Discarding the truncated reads that were shorter than 50 bp. (3) Reads which could not be assembled were discarded. OTUs were clustered with 97% similarity cutoff using UPARSE(version 7.1, http://drive5.com/uparse/) (Edgar 2013) and chimeric sequences were identified using UCHIME (Edgar et al. 2011). The phylogenetic affiliation of each 16S rRNA gene sequence was analyzed by the Ribosomal Database Project Classifier (RDP. Available online: http://rdp.cme.msu.edu/) using confidence threshold of 70% (Amato et al. 2013).

### Statistical analysis

All statistical analysis was calculated with SPSS 21.0 (SPSS, Inc., Chicago, IL). The data were normally distributed and were expressed as mean ± standard deviation (SD). Statistical differences in body weight and glucose metabolism parameters were determined using ANOVA followed by multiple‐comparison testing using Bonferroni post hoc analysis. PCA is an ordination method based on multivariate statistical analysis that maps the samples in different dimensions. Metastats was used to compare the relative abundance of each taxon at different taxonomic levels between groups. In addition, correlation analyses between the relative abundance of sequences belonging to different bacterial class and the AUC of IPGTT were performed by using Spearman's correlation analyses. *P* value <0.05 were considered to be statistically significant.

## Results

### Effects of catch‐up growth on offspring body weight

**Figure 1:**
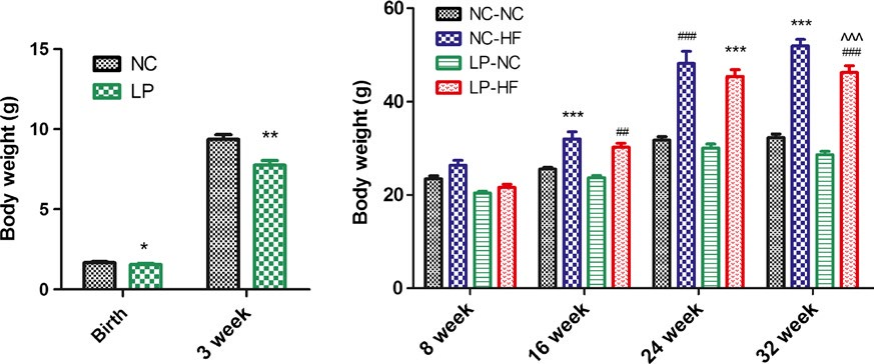
Maternal and postweaning imbalanced diets affected offspring body weight. Data represent as mean ± SD (*n* = 8, in each group). *P < 0.05, **P < 0.01, ***P < 0.001 NC‐ HF versus NC‐NC group. ^#^ *P *< 0.05, ^##^ *P *< 0.01, ^###^ *P *< 0.001 LP‐HF versus NC‐NC group. ^^^^^ *P *< 0.001 NC‐HF versus LP‐HF group. Diet abbreviations: NC, Normal chow; LP, Low protein; HF, High fat. Dam and pup diets denoted before and after the dash line, respectively.

Birth weight and body weight at weaning were both significantly lower in the offspring of the dams fed low‐protein (LP) diet (*P *< 0.05 and *P *< 0.01, respectively). However, after 5 weeks, there was no significant difference of body weight among the four groups (*P* > 0.05). Moreover, the offspring from normal chow (NC) and LP dams fed high fat (HF) diet from weaning had higher body weight at 16 weeks of age until to termination, compared with the NC‐NC offspring (*P *< 0.05). The body weight is heavier in NC‐HF offspring at 32 weeks of age than in LP‐HF offspring, but the difference is much smaller than difference between LP‐HF and NC‐NC offspring (Fig. 1).

### Effect of catch‐up growth on glucose tolerance in the offspring

**Figure 2:**
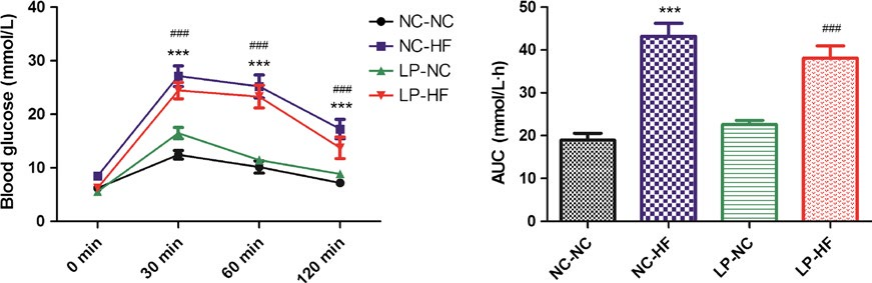
Maternal and postweaning imbalanced diets affected glucose tolerance and insulin sensitivity in the offspring. IPGTT(A) and area under the curve (AUC) (B). Data represent as mean ± SD (*n* = 8, in each group). ***P < 0.001 NC‐HF versus NC‐NC group, ^###^ *P *< 0.001 LP‐HF versus NC‐NC group. IPGTT: intraperitoneal glucose tolerance test. AUC: area under the curve. Diet abbreviations: NC, Normal chow; LP, Low protein; HF, High fat. Dam and pup diets denoted before and after the dash line, respectively.

The blood glucose levels of the male offspring in the NC‐HF and LP‐HF groups were significantly higher at 30 min (*P* < 0.001), 60 min (*P* < 0.001), and 120 min (*P* < 0.01) after intraperitoneal glucose administration, compared with those of the NC‐NC offspring. Consistently, the blood glucose AUC was significantly greater in NC‐HF and LP‐HF than NC‐NC offspring (*P* < 0.001) (Fig. 2).

### Effect of catch‐up growth on insulin sensitivity in the offspring

**Figure 3:**
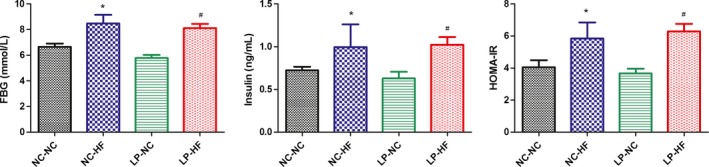
Maternal and postweaning imbalanced diets affected insulin sensitivity in the offspring. FBG(A), Serum insulin levels(B) and HOMA‐IR(C). Data represent as mean ± SD (*n* = 8, in each group). *P < 0.05 NC‐HF versus NC‐NC group, ^#^ *P *< 0.05 LP‐HF versus NC‐NC group. Area under the curve (AUC): area under the curve, IPGTT: intraperitoneal glucose tolerance test. FBG: Fasting blood glucose, HOMA‐IR: Homeostasis model assessment of insulin resistance. Diet abbreviations: NC, Normal chow; LP, Low protein; HF, High fat. Dam and pup diets denoted before and after the dash line, respectively.

We further examined the insulin sensitivity of the offspring. It indicated that both the fasting blood glucose (*P* < 0.05) and serum insulin concentrations (*P* < 0.05) of the NC‐HF and LP‐HF offspring were significantly higher than NC‐NC offspring at 32 weeks of age. Furthermore, HOMA‐IR of the NC‐HF and LP‐HF offspring was also significantly higher compared with the NC‐NC offspring (Fig. 3).

### Effect of catch‐up growth on lipid levels in the offspring

**Figure 4:**
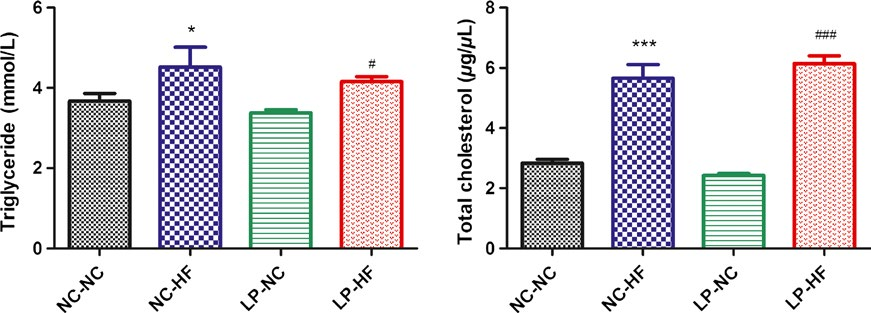
Maternal and postweaning nutrition imbalance induces dyslipidemia in the offspring. Serum triglyceride (A) and total cholesterol levels (B). Data represent as mean ± SD (n = 8, in each group). *P < 0.05, ***P < 0.001 NC‐HF versus NC‐NC group. ^#^ *P *< 0.05, ^###^ *P *< 0.001 LP‐HF versus NC‐NC group. Diet abbreviations: NC, Normal chow; LP, Low protein; HF, High fat. Dam and pup diets denoted before and after the dash line, respectively.

Serum triglyceride and total cholesterol levels were both significantly elevated in the NC‐HF (*P* < 0.05 and *P* < 0.001, respectively) and LP‐HF offspring (*P* < 0.05 and *P* < 0.001, respectively) at 32 weeks of age, compared with the NC‐NC offspring (Fig. 4).

### Lower bacterial richness and evenness in the offspring

Because there is no significant difference in body weight and all the glucose metabolism parameters between LP‐NC and NC‐NC groups throughout the experiment, we did not perform microbial diversity analysis for LP‐NC offspring. The microbial diversity of offspring in NC‐NC, NC‐HF, and LP‐HF groups (*n* = 6, each group) were investigated. A total of 245,220 high‐quality sequences were obtained in this study, with an average of 20,435 sequences per sample. The number of sequences was similar between the LP‐HF and NC‐NC groups (20,450 ± 3939 vs. 20,420 ± 5072, *P* > 0.05). The Good's coverage of each group was over 97%, indicating that the 16 S rRNA gene sequences identified can represent the majority of bacteria present in the samples of this study. The operational taxonomic units (OTUs), the estimators of community richness (Chao), and diversity (Shannon) are shown in Table 1. The Shannon's diversity index considers both richness and evenness. There were statistically significant differences in OTUs (131.70 ± 94.85 vs. 235.00 ± 8.19, *P* < 0.05) and Shannon indexes (2.96 ± 0.31 vs. 3.73 ± 0.09, *P* < 0.05) between LP‐HF and NC‐NC offspring. There were also statistically significant differences in OTUs (235.00 ± 8.19 vs. 110.00 ± 67.10, *P* < 0.05) and Shannon indexes (3.73 ± 0.09 vs. 2.67 ± 0.77, *P* < 0.05) between NC‐HF group and NC‐NC group, demonstrating the significantly lower bacterial richness and evenness found in NC‐HF and LP‐HF offspring compared with NC‐NC offspring (Table 1).

**Table 1:** Sequencing data summary and diversity analysis in the offspring

	NC‐NC	NC‐HF	LP‐HF
Sequences	20,420 ± 5072	21,826 ± 2532	20,450 ± 3939
OTUs	235.00 ± 8.19	110.00 ± 67.10^a^	131.70 ± 94.85^b^
Chao	257.30 ± 10.12	130.60 ± 62.10	147.00 ± 96.69
Shannon	3.73 ± 0.09	2.67 ± 0.77^a^	2.96 ± 0.31^b^

OTUs: operational taxonomic units Diet abbreviations: C, Control diet; HF, High fat diet. Dam and pup diets denoted before and after the dash line, respectively.

Data represent as mean ± SD (*n* = 6, in each group). The number of OTUs, richness estimator Chao, and diversity estimator Shannon were calculated at 3% distance.

a *P *< 0.05 NC‐HF versus the NC‐NC group.

b *P *< 0.05 LP‐HF versus the NC‐NC group.

### Differences in the microbial compositions in the offspring based on principal coordinates analysis (PCA) and Venn diagram

**Figure 5:**
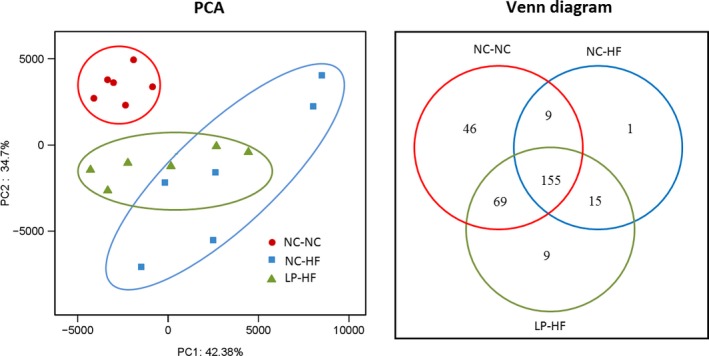
Differences in the microbial compositions in the offspring based on principal coordinates analysis(PCA) and Venn diagram. *n* = 6, in each group. Diet abbreviations: NC, Normal chow; LP, Low protein; HF, High fat. Dam and pup diets denoted before and after the dash line, respectively.

Principal coordinates analysis (PCA) of Illumina MiSeq amplicon data demonstrated significantly separate clustering of the gut microbial communities between the LP‐HF and NC‐NC offspring and NC‐HF also clustered away from the NC‐NC. Precisely, PC1 percent variation explained 57.54% and PC2 percent variation explained 22.63% (Fig. 5). It is indicated that there are significant differences in the microbial compositions between the groups. Venn diagram also revealed that the microbial community structure was significantly different in the offspring of LP‐HF and NC‐NC groups. More precisely, the Venn diagram showed that 224 OTUs were common in LP‐HF and NC‐NC offspring. Fifty‐five distinct OTUs were present only in NC‐NC offspring, whereas the other 24 OTUs were present only in the LP‐HF offspring (Fig. 5).

### Microbial structures in the offspring differed significantly

**Figure 6:**
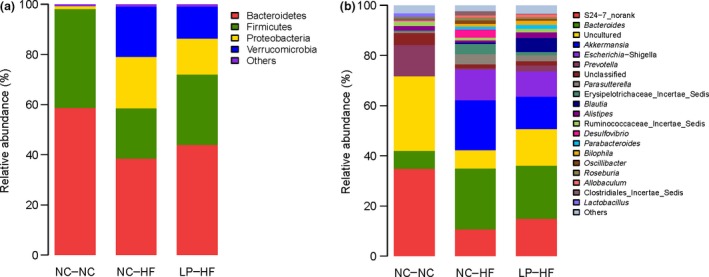
Relative abundance of bacterial phyla in microbiota at the phylum(A) and genus(B) level in the offspring. *n* = 6, in each group. Diet abbreviations: NC, Normal chow; LP, Low protein; HF, High fat. Dam and pup diets denoted before and after the dash line, respectively.

**Figure 7:**
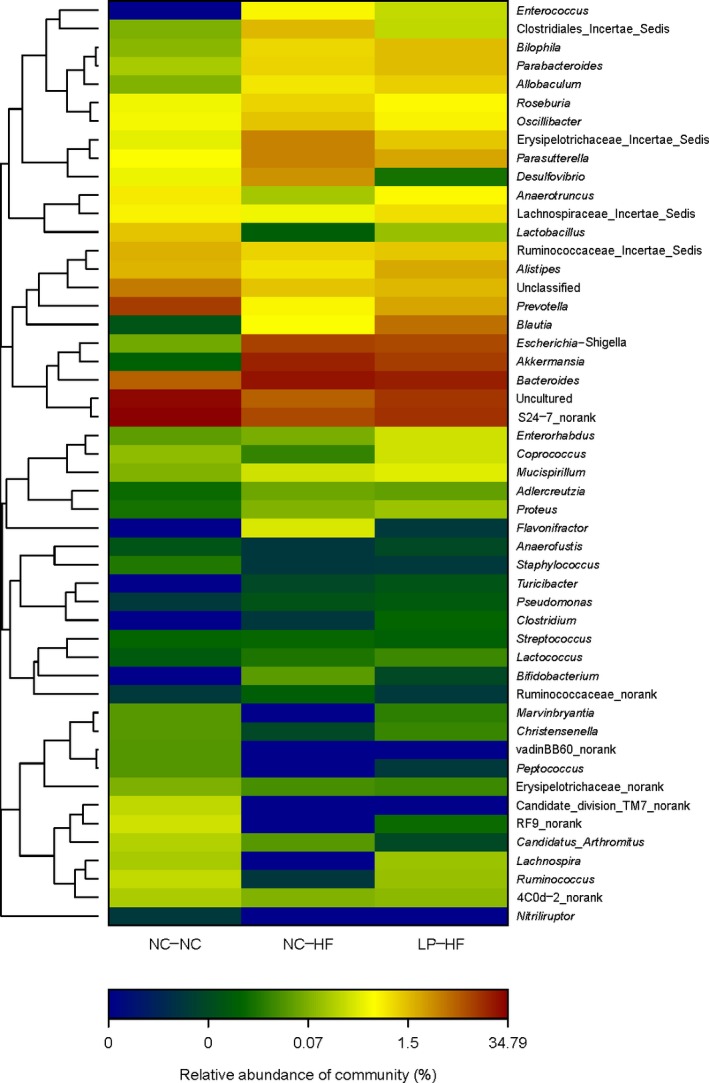
Heatmap analysis of abundant genera in the offspring. *n* = 6, in each group. The y axis is a neighbor‐joining phylogenetic tree, each row is a different phylotype. The abundance plot shows the proportion of 16S rRNA gene sequences in each group. Diet abbreviations: NC, Normal chow; LP, Low protein; HF, High fat. Dam and pup diets denoted before and after the dash line, respectively.

Figure 6 shows the overall microbiota structure for each group at the phylum and genus level in the LP‐HF and NC‐NC offspring. The dominant phyla of the LP‐HF and NC‐NC offspring were *Bacteroidetes*,* Firmicutes,* and *Proteobacteria*. *Bacteroidetes* were significantly decreased in the offspring from LP dams and weaned to HF diet. However, the *Verrucomicrobias* and *Proteobacteria* were significantly increased in LP‐HF offspring, compared with NC‐NC offspring (Fig. 6A). Figure 6B revealed that the genus‐specific relative abundance of microbiota were significantly different between the LP‐HF and NC‐NC offspring. The heatmap according to bacterial genus level also demonstrated the same phenomenon (Fig. 7).

### Effects of catch‐up growth on phylotypes in the offspring

There were significant variations in the composition of gut microbiota between the LP‐HF and NC‐NC offspring at different bacterial levels. More specifically, at the phylum level, the percentage of *Tenericutes*,*Candidate_division_TM7,* and *Bacteroidetes* was significantly lower in LP‐HF offspring. Conversely, *Verrucomicrobia* and *Proteobacteria* exhibited a statistically significant higher percentage in LP‐HF compared with NC‐NC offspring (*P* < 0.05). The microbial composition was also significantly different at the genus level, with nine genera being different between the LP‐HF and NC‐NC offspring. *Lactobacillus*,* RF9_norank*,* Candidate_division_TM7_norank*,* S24‐7_norank*,* vadinBB60_norank*,* Candidatus_Arthromitus,* and *Desulfovibrio* genera showed a significant lower percentage in LP‐HF offspring (*P* < 0.05), whereas *Akkermansia* and *Parabacteroides* genera demonstrated significant higher percentage in LP‐HF, compared with NC‐NC offspring (*P* < 0.05). *Lactobacillus genus* exhibited a statistically significant higher percentage in LP‐HF compared with NC‐HF offspring*,* whereas *Erysipelotrichaceae_Incertae_Sedis* showed a significant lower percentage in LP‐HF compared with NC‐HF offspring (*P* < 0.05; Table 2).

**Table 2:** Significant variations in the compositions of gut microbiota at the phylum and genus level in the offspring

Taxonomic rank	Feature	NC‐NC (%)	NC‐HF (%)	*P* value^a^	LP‐HF(%)	*P* value^b^	*P* value^c^
Phylum	*Bacteroidetes*	57.47	38.61	0.03	43.26	0.04	–
Phylum	*Firmicutes*	40.68	20.69	0.04	28.03	–	–
Phylum	*Proteobacteria*	1.13	20.07	–	14.63	0.04	–
Phylum	*Tenericutes*	0.26	0.00	0.00	0.01	0.01	–
Phylum	*Candidate_division_TM7*	0.18	0.00	0.02	0.00	0.03	–
Phylum	*Verrucomicrobia*	0.01	19.63	–	13.32	0.02	–
Genus	*S24‐7_norank*	34.69	10.78	0.00	13.36	0.03	–
Genus	*Prevotella*	11.51	0.70	0.03	2.03	–	–
Genus	*Bacteroides*	7.14	24.29	0.02	22.65	–	–
Genus	*Lactobacillus*	1.50	0.01	0.00	0.12	0.00	0.04
Genus	*RF9_norank*	0.26	0.00	0.00	0.01	0.00	–
Genus	*Candidate_division_TM7_norank*	0.18	0.00	0.01	0.00	0.01	–
Genus	*vadinBB60_norank*	0.04	0.00	0.03	0.00	0.04	–
Genus	*Parabacteroides*	0.14	1.13	0.04	1.74	0.04	–
Genus	*Ruminococcus*	0.22	0.00	0.01	0.12	–	–
Genus	*Christensenella*	0.04	0.00	0.02	0.02	–	–
Genus	*Coprococcus*	0.09	0.02	0.02	0.23	–	–
Genus	*Erysipelotrichaceae_Incertae_Sedis*	0.46	4.31	0.02	1.30	–	0.04
Genus	*Akkermansia*	0.01	19.63	–	13.32	0.01	–
Genus	*Candidatus_Arthromitus*	0.17	0.04	–	0.00	0.04	–
Genus	*Desulfovibrio*	0.41	3.39	–	0.01	0.04	–

Diet abbreviations: C, Control diet; LP, Low‐protein diet; HF, High fat diet. Dam and pup diets denoted before and after the dash line, respectively.

*P* value had no statistically significant difference (≥0.05) were not shown.

a *P *< 0.05 NC‐HF versus the NC‐NC group.

b *P *< 0.05 LP‐HF versus NC‐NC group.

c *P *< 0.05 LP‐HF versus NC‐HF group.

### Correlation analysis

**Figure 8:**
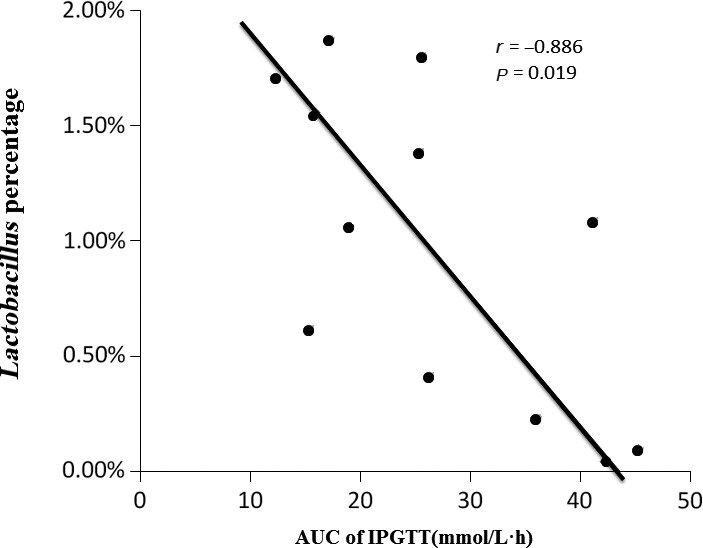
Correlation analysis between relative abundance (%) of bacterial genus and blood glucose concentrations of IPGTT. *n* = 18. Area under the curve (AUC): area under the curve, IPGTT: intraperitoneal glucose tolerance test. Diet abbreviations: NC, Normal chow; LP, Low protein; HF, High fat. Dam and pup diets denoted before and after the dash line, respectively.

In order to evaluate the relationship between gut microbial communities and glucose metabolism in the offspring, correlation analyses between the relative abundance (%) of sequences belonging to a specific bacterial genus in all the offspring and the glucose response to a glucose load were performed. It suggested that *Lactobacillus* percentage was negatively associated with AUC of IPGTT (*r* = −0.886, *P* = 0.019) (Fig. 8).

## Discussion

The developmental origins of health and disease (DOHaD) hypothesis predicts that environmental exposures such as nutrition experienced early in life have the potential to modify susceptibility to later‐onset metabolic diseases (Saffery 2014). In this study, we showed that impaired nutrient supply during perinatal growth‐induced adaptive changes, leading to metabolic disorders, such as obesity, impaired glucose tolerance, insulin resistance, dyslipidemia, and nonalcoholic fatty liver disease when the organism was challenged with increased caloric intake. Our results are consistent with previous studies showing that maternal imbalanced nutrition‐induced detrimental consequences on glucose and lipid homeostasis (Claycombe et al. 2013; Baik et al. 2014; De Toro‐Martin et al. 2014; Sellayah et al. 2014). Therefore, maternal undernutrition results in lower birth weight that is accompanied by the development of chronic metabolic diseases in later life.

Numerous studies demonstrated that the microbiota could be considered as one major player in the development of obesity, diabetes mellitus, and nonalcoholic fatty liver disease (Ramakrishna 2013; Gangarapu et al. 2014). Therefore, we investigated the “programming” effects of maternal nutrition imbalance on gut microbial compositions in the offspring. Our studies indicated that the offsprings that experienced either a maternal protein restriction or normal chow diet followed by high‐energy nutrition had significantly lower bacterial richness and evenness. Similarly, one large clinical study also showed that obesity, insulin resistance, and fatty liver were more prevalent in individuals with low bacterial richness compared with subjects characterized by high gene count (Le Chatelier et al. 2013).

In agreement with previous studies, our study showed that the dominant phyla of all the offspring were *Bacteroidetes*,* Firmicutes*, and *Proteobacteria* (Karlsson et al. 2011; Evans et al. 2014). We herein showed that *Bacteroidetes* were significantly decreased in the offspring of nutrition‐induced catch‐up growth. Evidence in the literature also indicated that the levels of *Bacteroidetes* in the gut microbiome were reduced with obesity while elevated when obese individuals lose weight (Turnbaugh et al. 2009). In another study, it was observed that there was a lower abundance of *Bacteroidetes* in nonalcoholic steatohepatitis patients compared with healthy control subjects (Mouzaki et al. 2013).

Furthermore, our study showed *Lactobacillus* percentage was negatively associated with blood glucose concentrations of IPGTT. This indicated gut microbiota communities such as *Lactobacillus* might be an important bacterial phylum in the metabolic programming of nutrition‐induced catch‐up growth. Several studies also showed that probiotic products could regulate blood glucose levels in diabetic human and *Lactobacilli* are often used as probiotic agents (Ejtahed et al. 2012). One previous study indicated that probiotic yogurt containing *Lactobacillus acidophilus La5* and *Bifidobacterium lactis Bb12* significantly decreased fasting blood glucose and hemoglobin A1c and increased erythrocyte superoxide dismutase in patients with type 2 diabetes mellitus (Ejtahed et al. 2012). Naito et al. reported that oral administration of *Lactobacillus casei strain Shirota* had the potential to prevent obesity‐associated metabolic abnormalities by improving insulin resistance in diet‐induced obesity mice (Naito et al. 2011). A recent study also demonstrated that *Lactobacillus casei* could markedly prevent rats from the onset of type 2 diabetes (Zhang et al. 2014). However, various Lactobacillus species have also been reported as positively correlated with glucose intolerance in several independent clinical studies (Delzenne et al. 2015). One study showed that Lactobacillus species correlated positively with fasting glucose and HbA1c levels (Karlsson et al. 2013). Other studies also showed that bacteria increased in the gut of type 2 diabetic patients also included the sulfate‐reducing bacteria Desulfovibrio, as well as Lactobacillus gasseri, Lactobacillus reuteri, and Lactobacillus plantarum (Sato et al. 2014). Together, these data indicate that *Lactobacillus* may play an important to modulate gut microbiota and the impact of Lactobacillus on glucose metabolism is still controversial.

## Conclusions

This study clearly shows that nutrition‐induced catch‐up growth predisposes the offspring to obesity, impaired glucose tolerance, insulin resistance, nonalcoholic fatty liver disease, and gut microbiota perturbation in later life. Our work is novel in showing the “programming” effects of maternal nutrition imbalance on gut microbiota and metabolic diseases in adult mice. Therefore, it is suggested that therapeutic interventions targeting gut microbiota may be a new theoretical basis for preventing the onset of such metabolic diseases in individuals with previous episodes of growth restriction.

## Conflicts of Interest

The authors declare no conflict of interest.
